# Intraoperative bronchospasm with thiopental

**DOI:** 10.4103/0019-5049.63646

**Published:** 2010

**Authors:** Aparna Shukla

**Affiliations:** Department of Anaesthesiology, Era's Lucknow Medical College and Hospital, Lucknow, India

Sir,

Induction of anaesthesia with thiopental sometimes leads to bronchospasm especially in patients with a history of bronchial asthma. Although bronchospasm with thiopental is a rare phenomenon, it can be catastrophic if it occurs. Mechanism of bronchospasm with thiopental can be manifold. We report a case of severe intraoperative bronchospasm following induction of anaesthesia with thiopental.

A 24-year-old female presented in emergency department with breast abscess, for emergency incision and drainage procedure. The patient gave a history of childhood asthma but was completely alright for the last 15 years. The patient also gave a history of few seizure episodes in past for which she did not take any treatment. In the emergency ward intravenous line was established and intravenous infusion with DNS started. Patient was kept nil orally for 6 hours. Baseline investigations were ordered. In the meantime, inj. Tramadol 100 mg i.v. was given to the patient for the relieving pain. She also received inj. Ranitidine 150 mg i.v. and inj. metoclopramide 10 mg i.v. On arriving at the operation theatre, the patient was premedicated with inj. Midazolam 1.5 mg i.v. and inj. Fentanyl 100 mcg i.v. General anaesthesia with endotracheal intubation was planned. After preoxygenation for 3 minutes, induction was done with inj. thiopental in doses of 5 mg/kg. Immediately after that, the patient developed sudden spasm and it became almost impossible to ventilate the patient with bag and mask. SpO_2_ started falling and gradually dropped to 80%. On auscultation, wheezing was heard all over the chest. Without wasting much time, inj. Succinylcholine 75 mg was given i.v. and the patient was intubated with cuffed Endotracheal tube 7.5 mm. Bain circuit was connected and the patient was ventilated with 100% oxygen. This was followed by inj. Hydrocortisone 200 mg i.v. and inj. Dexamethasone 8 mg i.v. Aminophylline bolus was given in doses of 5 mg/kg followed by infusion in drip. After about 3–4 minutes, SpO_2_ started improving, and on auscultation, the wheezing disappeared. Surgery was allowed to proceed thereafter. The patient was maintained on O_2_ (40%), N_2_O (60%) and halothane (0.8%) on controlled ventilation till spontaneous ventilation was resumed. After completion of the procedure, the patient was extubated and shifted to preoperative ward after monitoring for a suitable period. On the next day, the patient was discharged from the hospital.

Bronchospasm with thiopental is a rare occurrence. Many theories have been proposed for this but none of them has been comprehensively defined. Direct spastic effect of thiopental on bronchial smooth muscle,[1,2] release of histamine and cholinergic stimulation[3] have been proposed as a mechanism of spasm with thiopental. Even insertion of laryngeal mask airway under light thiopental anaesthesia may evoke sudden bronchospasm.[4]

In our case, as the patient had a history of seizure attack, we avoided using ketamine. Propofol has a controversial role in epileptic patients. Some recommended caution in administering Propofol in epileptic patients.[5] Hughes and Lyons reported prolonged myoclonus associated with meningismus after administering Propofol.[6]

In this particular case, apart from falling SpO_2_, the patient did not have any another symptoms pertaining to histamine release; therefore, chances of anaphylactic reactions were minimal. Because the patient already had tachycardia and we did not use cholinergic premedication, one cause of spasm with thiopental in our case might be cholinergic nerve stimulation with the drug in question. Another cause might be the direct effect of thiopental on bronchial smooth muscle.

In conclusion, we would like to stress upon the fact that although bronchospasm with thiopental is a rare phenomenon it cannot be overlooked. Therefore, it is recommended that thiopental should be avoided or used cautiously in patients with a history of asthma.
